# Characteristics of exposure to radioactive iodine during a nuclear incident

**DOI:** 10.2478/raon-2024-0051

**Published:** 2024-10-04

**Authors:** Katja Zaletel, Anamarija Mihovec, Simona Gaberscek

**Affiliations:** Division of Nuclear Medicine, University Medical Centre Ljubljana, Ljubljana, Slovenia; Faculty of Medicine, University of Ljubljana, Ljubljana, Slovenia

**Keywords:** thyroid, radioactive iodine, nuclear accident, thyroid cancer, potassium iodide

## Abstract

**Background:**

During a nuclear accident, numerous products of nuclear fission are released, including isotopes of radioactive iodine. Among them is iodine-131, with a half-life of 8.02 days, which emits β radiation. For decades, it has been effectively and safely used in medicine. However, in the event of a nuclear accident, uncontrolled exposure can have harmful biological effects. The main sources of internal contamination with iodine-131 are contaminated air, food and water. The most exposed organ is the thyroid gland, where radioactive iodine accumulates via the Na+/I− symporter (NIS). NIS does not distinguish between radioactive iodine isotopes and the stable isotope iodine-127, which is essential for the synthesis of thyroid hormones. Exposure to radioactive iodine during a nuclear accident is primarily associated with papillary thyroid cancer, whose incidence begins to increase a few years after exposure. Children and adolescents are at the highest risk, and the risk is particularly significant for individuals living in iodine-deficient areas.

**Conclusions:**

Ensuring an adequate iodine supply is therefore crucial for lowering the risk of the harmful effects of exposure to radioactive iodine at the population level. Protecting the thyroid with potassium iodide tablets significantly reduces radiation exposure, as stable iodine prevents the entry of radioactive iodine into the thyroid. Such protection is effective only within a narrow time window - a few hours before and after the exposure and is recommended only for those under 40 years of age, as the risks of excessive iodine intake outweigh the potential benefits in older individuals.

## Introduction

Various sources of ionizing radiation play a crucial role in nuclear medicine, industry, the military, as well as in science and research. Nuclear power plants, significant sources of electrical energy, exploit the nuclear fission reaction of enriched uranium-235 or plutonium-239. Risks associated with radioactive contamination in the event of a nuclear reactor accident have been the subject of numerous public debates, especially in the last few decades following the catastrophic consequences of the accidents in Chernobyl in 1986 and Fukushima in 2011.^1,2^

The nuclear fission reaction was also characteristic of nuclear weapons used in the Second World War. A representative of the newer generation of nuclear weapons is the hydrogen bomb, which utilizes the process of nuclear fusion in combination with nuclear fission and can be up to 1000 times more powerful than a fission bomb.^3^ In addition to the threat of nuclear warfare, nuclear terrorism poses one of the major threats to international security today. It involves the illegal and intentional use of radioactive material to achieve various harmful objectives. This includes terrorist attacks on nuclear power plants and the use of nuclear weapons, as well as the use of “dirty bombs” that disperse radioactive substances into the environment without a nuclear explosion.^4^

**FIGURE 1.**
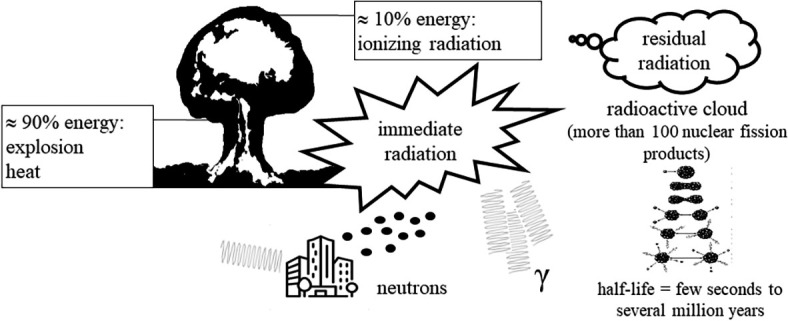
Sources of ionizing radiation during a nuclear accident.

According to the definition provided by the International Atomic Energy Agency, nuclear and radiation accidents involve exposure to radioactive radiation, resulting in significant consequences for individuals, the environment, or objects.^5^ In contrast to radiation incidents, where exposure to radioactive radiation is not linked to nuclear fission, nuclear accidents are distinguished by their association with an explosion that involves nuclear fission. This can be observed in events such as a nuclear bomb detonation or a nuclear reactor incident.^4,6^ Such accidents are characterized by a substantial release of energy, with approximately 90% being released in the form of explosion and heat, and about 10% being released in the form of ionizing radiation. Additionally, a variety of nuclear fission products are released, including isotopes of radioactive iodine (Figure 1).^7^

## Sources of ionizing radiation in a nuclear incident

In a nuclear accident, energy in the form of ionizing radiation is predominantly released immediately within the first minute after the explosion. The risks associated with immediate radiation primarily relate to the harmful effects of gamma and neutron radiation, which have the highest penetration capability.^8,9^ Neutron radiation, in addition to its direct effects on living organisms, destabilizes stable atoms of materials (such as iron and concrete) in objects surrounding the explosion, transforming them into new sources of ionizing radiation. Over an extended period following the explosion, residual radiation is emitted into the atmosphere in the form of a radioactive cloud, traveling several hundred kilometers from the accident site, and depositing radioactive substances gradually onto the Earth (Figure 1).^9^

In the immediate vicinity of the explosion site, larger radioactive particles settle locally, with the most intense settling occurring within the first 24 hours. Smaller particles reaching the troposphere continue to settle for several months after the accident, particularly in the broader vicinity of the nuclear explosion. The smallest particles, especially in powerful nuclear weapon explosions, can reach the stratosphere, settling on the entire surface of the Earth for several years after the explosion.^4,9^

During a nuclear accident, a broad spectrum of different radioactive fission products can be produced, with half-lives ranging from a few seconds to several million years.^7,10^ Their total radioactivity is initially extremely high, but it decreases relatively rapidly due to radioactive decay.^11^ Only those radioactive isotopes with appropriate physical properties (small particles reaching higher atmospheric layers, water-soluble particles, etc.) and a sufficiently long half-life can represent a long-term source of radiation exposure in the broader vicinity of a nuclear incident. Examples of such radioactive isotopes that are a source of harmful β radiation include cesium-137, strontium-90, and iodine-131.^12,13^ Similar to the mentioned isotopes, xenon-133 is also a source of β radiation, easily entering the atmosphere due to its gaseous form. Although its physical half-life is approximately 5 days, its biological half-life is only 30 seconds. After entering the body, it is exhaled within a few minutes, thus having no significant harmful effects.^10^

Cesium-137, with a half-life of approximately 30 years, has a relatively low boiling point and is water-soluble. Consequently, it travels effectively in the air, spreading even after deposition from the atmosphere to the soil, causing radioactive contamination of land, water, and living organisms. Once absorbed into the body, it accumulates in tissues, constituting a source of prolonged exposure to radiation.^12^ Strontium-90, with a half-life of 28 years, chemically resembles calcium. As a result, it accumulates in bones and teeth, representing a source of radiation exposure for the bone marrow.^14^ Iodine-131 is water-soluble and emits both β radiation and, to a lesser extent, γ radiation. Compared to cesium-137 and strontium-90, it has a significantly shorter half-life, causing no long-term environmental contamination. It accumulates in the thyroid gland, where it has harmful biological effects.^10,15^

## Characteristics of iodine isotopes

There are 37 known isotopes of iodine, ranging from iodine-108 to iodine-144. The only stable isotope is iodine-127, which is essential for the synthesis of thyroid hormones and is commonly consumed in the form of iodized salt in everyday life. All other iodine isotopes exhibit radioactive decay with half-lives, mostly shorter than 60 days. Only iodine-129 has a long half-life of 1.57 × 10^7^ years.^16^

In medicine, radioactive iodine has been used for several decades, particularly for diagnosing and treating thyroid diseases.^17^ Various isotopes of iodine, including iodine-123, iodine-124, iodine-125, and iodine-131 play important roles today. Iodine-123 is a cyclotron-produced isotope with a half-life of 13.2 hours. It emits low-energy γ radiation with a long range, causing no tissue destruction. It is suitable for diagnostic purposes, as the γ radiation detected by a gamma camera provides valuable information about the uptake of iodine in the thyroid.^18^ Similarly, iodine-124 is a cyclotron-produced isotope with a half-life of 4.18 days. Due to the emission of positrons during radioactive decay, it is suitable for imaging with positron emission tomography.^19^ Iodine-125, obtained in nuclear reactors, has a long half-life of 59.4 days and emits low-energy γ radiation. It is used in brachytherapy^20^ and serves as a tracer in radioimmunoassays for the laboratory determination of various analytes.^19^

Iodine-131, also obtained in nuclear reactors, has a half-life of 8.02 days. Upon decay, it emits high-energy β radiation of 0.61 MeV with a short tissue range of up to 0.8 mm.^19,21^ Iodine-131 is the treatment of choice for patients with autonomous thyroid tissue and a second-line treatment for patients with Graves' disease. In both patient groups, the goal of treatment is to alleviate hyperthyroidism. Iodine-131 is an effective medication for ablating residual thyroid tissue after thyroid cancer surgery, and it can also be used to treat euthyroid nodular goiter with the goal of reducing thyroid volume.^21^ The activity required for the successful treatment of thyroid diseases must be sufficiently high to expose the target tissue to the deterministic effects of iodine-131. Our study involving patients with Graves' disease, for example, indicates that iodine-131 treatment successfully eliminated hyperthyroidism in over 90% of patients with an average received dose of 144 Gy or 164 Gy, whereas in patients with an average received dose of 105 Gy, success was achieved in only 64% of patients.^22^

## Iodine and the thyroid

Non-radioactive or stable iodine is a fundamental constituent element of thyroid hormones thyroxine (T4) and triiodothyronine (T3), which are essential for metabolism in all age groups and for the develpoment and brain maturation in foetuses and young children. According to World Health Organisation (WHO) recommendations, the daily iodine intake for adults should be around 150 μg, while pregnant and lactating women should aim for around 250 μg.^23^

A healthy adult body contains 15–20 mg of iodine, 70–80% of which is stored in the thyroid gland.^24^ As reported, serum concentration of free iodide (I^−^), however, is only 50 nM to 300 nM.^25^ The thyroid cells have evolved an extremely efficient mechanism to accumulate iodine. The glycoprotein responsible for active iodine transport into the thyroid cell was identified in 1996 as Na^+^/I^−^ symporter (NIS), localized in the basolateral membrane of thyroid epithelial cells, facing the bloodstream.^26,27^ NIS facilitates Na^+^/I^−^ symport with a 2:1 stoichiometry, driven by the Na^+^ electrochemical gradient established by the basolateral Na^+^/K^+^ ATPase. As a result, I^−^ is actively concentrated in the thyroid cells. NIS cannot differentiate between stable and radioactive iodine, making it a powerful tool for diagnostics and treatment with radioiodine, as it rapidly concentrates in the thyroid.^27^ Upon entering the thyroid cell, I^−^ passes transcellularly to reach the apical membrane. Here, it undergoes oxidation catalysed by the enzyme thyroid peroxidase (TPO) in the presence of H2O2, followed by iodination of tyrosine residues on thyroglobulin (Tg) and synthesis of thyroid hormones.^28^

The regulation of NIS is primarily influenced by thyroid stimulating hormone (TSH), a pituitary hormone. TSH, a key regulator of thyroid function and size, stimulates thyroid gland by promoting NIS transcription, upregulating the expression of TPO and Tg, as well as facilitating Tg endocytosis. Moreover, TSH also regulates NIS localization and is necessary for targeting NIS to the plasma membrane, as well as its retention there.^29^

In addition to TSH, iodine content in the thyroid cell itself regulates the I^−^ uptake. If iodine content is low, the expression of NIS is increased and vice versa. A mechanism, known as autoregulation, enables the normal synthesis of thyroid hormones irrespective of iodine supply.^30^ Exposure to high concentrations of I^−^ inhibits thyroid hormone synthesis and secretion, likely by supressing H_2_O_2_ production and reducing the expression of TPO and Tg. This phenomenon was named the Wolff-Chaikoff effect.^30,31^ However, despite ongoing excess of I^−^, its inhibitory effect diminishes after approximately 48 hours, allowing for the restoration of thyroid hormone synthesis. This escape from the Wolff-Chaikoff effect is enabled by an intrinsic autoregulatory mechanism, wherein NIS is down-regulated by high intracelluar I^−^ leading to the intracellular iodine concentration below critical inhibitory threshold.^31^ This downregulation occurs through several mechanisms, including inhibition of NIS transcription and increased degradation of NIS mRNA and NIS protein as well as translocation of NIS molecules from the basolateral membrane into the thyroid cell.^27,30^

## Radioactive iodine contamination in a nuclear accident

During a nuclear accident, the by-products of nuclear fission released into the environment include various isotopes of radioactive iodine. Notably, iodine-131, with its relatively long half-life and high energy, poses the most significant biological risks.^32^ Released in the form of a radioactive cloud, radioactive iodine contaminates air, water, soil, vegetation, and surfaces, thereby constituting a source of external contamination. Inhalation of contaminated air and ingestion of tainted food and water result in internal contamination of both humans and animals.^33^ For infants of exposed mothers, breastfeeding is also a risk factor for iodine-131 ingestion, since NIS expression in breast occurs during lactation enabling I^−^ secretion into the milk as the sole source of this nutrient for the newborn.^27^ During internal contamination, the thyroid is the most exposed organ, as approximately 10–30% of the incorporated amount accumulates in it within 24 hours, facilitated by the action of NIS. Most of the remaining radioactive iodine is excreted from the body with urine.^34^

Experiences from Chernobyl reveal that contaminated cow's milk was the primary source of iodine-131 internal contamination for residents, while the contaminated air affected exposed workers at the power plant. Factors such as age, place of residence, and milk consumption habits during the first 8 weeks after the accident had the greatest impact on the doses received by residents.^35^ They estimate that residents in exposed areas of Belarus and Ukraine received an average thyroid dose of about 0.65 Gy, with the maximum dose reaching 42 Gy. Workers at the power plant exposed to radioactive iodine received an average dose of 0.18 Gy. In the most affected region of Belarus, children received an average dose of 0.75 Gy, with a maximum estimated dose of 8.7 Gy.^36^ According to the largest study on in utero exposure to iodine-131 from Chernobyl fallout in selected regions of Ukraine, the mean estimated fetal thyroid dose was 0.072 Gy, with a range of 0–3.23 Gy.^37^ Higher thyroid doses in children and adolescents compared to adults are attributed to factors such as a higher iodine uptake, smaller thyroid glands, and greater milk consumption.^38^ A 5-year-old child at the time of the accident received a thyroid dose approximately four times larger than that of an adult.^39^

In Fukushima, only approximately 10% of radioactivity compared to Chernobyl was released.^40^ Early public notification prevented the majority of residents from ingesting contaminated water and food, making inhalation of iodine-131 the primary route of internal contamination.^41^ According to one of the earliest reports, the median thyroid dose was estimated at 0.0042 Gy for exposed children and 0.0035 Gy for adults.^42^ A recent assessment of children who were 1 year old at the time of the accident in the most affected areas around the Fukushima power plant showed that their thyroid glands were exposed to an average dose of 0.015 Gy, with the maximum received dose being 0.029 Gy.^43^ These values appear to be lower than earlier estimates, where average thyroid doses at 1 year ranged from 0.033 to 0.083 Gy.^44^ Among workers 0.7% exceeded thyroid dose of 0.1 Gy, while the majority received less than 0.1 Gy.^45^ Unlike the Chernobyl accident, where residents' thyroids were primarily exposed to iodine-131, in Fukushima, internal contamination with iodine-131 contributed to thyroid dose in 40–50%, with other short-lived isotopes of radioactive iodine (iodine-132, iodine-133, iodine-135) contributing 5–20%, and external irradiation due to radionuclides in the radioactive cloud and on surfaces in 40–50%.^41^

Among atomic bomb survivors from Hiroshima and Nagasaki exposed as children under 10 years, the mean thyroid radiation dose was 0.182 Gy, ranging from 0–4 Gy^46^, whereas the mean maternal uterine radiation dose was 0.256 Gy.^47^ After atmospheric nuclear weapons tests conducted in the second half of the last century in Arizona, Kazakhstan, China and French Polynesia, the mean estimated thyroid doses were up to 4 Gy due to radioactive fallout and external thyroid irradiation, whereas they were several times higher during experiments on the Marshall Islands.^44^

## Harmful effects of radioactive iodine in nuclear accident

The harmful effects of I-131 in a nuclear accident are primarily stochastic in nature, meaning they are random, with their likelihood proportional to the received dose, while the level of harm is not dependent on the dose size.^43^ They are usually associated with a higher incidence of papillary thyroid cancer and benign thyroid nodules, as well as a higher prevalence of autoimmune thyroid diseases.^33,44^ Low doses are typically classified as those under 0.1 Gy, while moderate doses fall within the range of 0.1 to 1 Gy.^47^ Exposure to high I-131 doses results in deterministic effects, where the frequency and severity increase with the dose after a threshold dose is reached, potentially resulting in hypothyroidism.^48^ Unlike the effects of uncontrolled exposure to I-131, in medicine we safely utilize its deterministic effects through the targeted, controlled use of higher activities of I-131 (Table 1).

**TABLE 1.** Differences in exposure to iodine-131 in medicine and during nuclear accident

**Parameter**	**In medicine**	**In nuclear accident**
**Radioactivity**	High	Low
**Average received dose (Gy)**	> 100	< 10
**Effects**	Deterministic	Stochastic
**The source**	Controlled production in a nuclear reactor	Uncontrolled release during a nuclear accident (nuclear reactor, nuclear bomb)
**Form**	Capsule Solution	Radioactive cloud
**Body intake**	Ingestion Intravenously	Ingestion Inhalation

The most vulnerable to the harmful effects of radioactive iodine are the thyroids of children, especially those under 5 years of age.^10^ Additionally, research has shown a significant inverse correlation between age at radiation exposure and thyroid cancer risk, with this correlation diminishing to statistical insignificance by age of 15.^49^ The increased cancer risk is attributed to rapid tissue growth and smaller thyroid sizes, resulting in higher radiation doses.^37,45^ Moreover, this elevated risk persists for at least four decades after exposure.^10,48,49^ Even doses as low as 0.05–0.1 Gy have been linked to higher thyroid cancer risk in children, with a linear dose-response up to about 10–20 Gy, beyond which the risk stabilizes.^44,48,49^ In individuals with radiation exposure in utero the risk of cancer is comparable to that of those exposed during childhood.^50^ The ability of the fetal thyroid to take up iodine increases from the third month, reaching the maximum at around the sixth month of pregnancy. During this period, the fetal thyroid receives the highest dose in cases of iodine-131 exposure.^51^ In early pregnancy, the fetal exposure originates from the iodine-131 activity in the mother's thyroid, peaking at one month of gestation and then gradually decreasing during gestation.^52^

Experiences from Chernobyl indicate that the incidence of thyroid cancer began to increase only 4–5 years after exposure. In the population under 18 years of age in 1986 residing in contaminated areas of Belarus, Ukraine and Russia, nearly 20,000 new cases of thyroid cancer were detected between 1991 and 2015.^35^ In individuals younger than 15 years who received a thyroid dose of ≥ 0.3 Gy, the risk of thyroid cancer was 5 times higher than in individuals with a received dose < 0.3 Gy.^39^ The Belarus data reveal distinctions in radiation-related pediatric thyroid cancers compared to radiation-nonrelated cases, including a higher incidence in boys, in children of the youngest age, a dominant follicular structural component, extrathyroidal tumor extension, and greater risk of distant metastases.^53,54^ However, the 15-year overall survival rate in radiation-related cases is excellent, exceeding 95%, despite recurrences occurring in 28% of cases.^53^ Childhood exposure of Belarus residents was also associated with benign thyroid nodules larger than 10 mm and the risk significantly increased with thyroid dose.^55^ In a cohort of exposed Ukrainian subjects with an estimated mean prenatal thyroid dose of 0.073 Gy, a markedly increased risk of thyroid cancer and a strong, significant dose-response relationship for large (≥ 10 mm) benign thyroid nodules were found three decades after the Chernobyl nuclear accident.^56^

After the Fukushima accident, a 10-year follow-up of individuals exposed before the age of 18, using ultrasound screening, confirmed a 10-fold increase in the prevalence of thyroid cancer, predominantly the papillary variant.^57,58^ Some believe that this observation might reflect overdiagnosis due to the use of highly sensitive ultrasound equipment during screening.^44,59^ However, analysis of a substantial number of operated patients revealed cervical lymph node metastases in 79% and extrathyroidal spread in 46%.^58^ Furthermore, a strong positive correlation was observed between the incidence rate of thyroid cancer among exposed children and thyroid dose, underscoring the necessity for close monitoring in high-risk individuals.^44,59^

During the follow-up of Japanese atomic bomb survivors, the increased thyroid cancer risk persisted for more than 50 years after childhood exposure, with about 36% of thyroid cancer cases being attributable to radiation exposure before age of 20.^60^

Hypothyroidism can be directly related to the deterministic effects of radiation, or it can be a result of autoimmunity induced by radiation exposure.^40^ After the Chernobyl accident, hypothyroidism was observed in 4.8% of emergency workers and in 3–6.2% of children under 18 years of age at the time of the accident.^40,61,62^ The risk of hypothyroidism increased with thyroid dose, decreased with increasing age at exposure and was similar for both genders.^62^ In Fukushima, where thyroid doses were much lower, the association between thyroid dose and hypothyroidism was not confirmed.^40^ More than six decades after the bombing, observations in atomic bomb survivors exposed as children, who had a mean thyroid radiation dose of 0.182 Gy, confirmed hypothyroidism in 7.8% and positive thyroid antibodies in 21.5%. None of these observations were associated with radiation dose.^45^

## The impact of iodine deficiency on the effects of exposure to radioactive iodine

One of the key factors regulating the uptake of iodine by the thyroid is the iodine supply. Adequate iodine supply for populations is ensured through national iodine fortification programs, with the iodization of table salt being the easiest and most effective method.^23,63^ Iodine deficiency is indeed associated with health complications, such as goitre and hypothyroidism. It leads to increased secretion of TSH, which stimulates the expression of NIS to maximize iodine uptake into thyrocytes.^64^

It was shown that after the improvement in iodine supply, thyroid uptake decreases.^65,66,67,68^ In Poland, an approximately 40% decrease in 24-hour iodine uptake was observed in euthyroid patients following a 30% increase in salt iodization.^65^ In Graves' patients a 40% decrease in radioiodine uptake was associated with a 74% increase in iodine intake.^66^ Twice the urinary iodine excretion was associated with a 25% lower iodine intake.^67^ Ten years after the 2.5-fold increase in mandatory salt iodization in Slovenia, the early and late thyroid uptake of iodine were significantly lower (37% and 32%, respectively) than before the increase.^68^ Most likely, the decrease in early thyroid uptake reflects decreased expression and activity of NIS.^69^ The mechanism for the decrease in late thyroid uptake could be increased intracellular iodine content, which decreases the incorporation of diagnostic radiopharmaceuticals into thyroid hormones.^68^

In accordance with thyroid uptake research findings, studies demonstrate that the improvement of iodine supply is also associated with a higher activity of iodine-131, needed for the successful treatment of thyroid diseases. In patients with Graves' disease, 40% higher iodine-131 activity was required to cure hyperthyroidism after a 74% increase in iodine intake.^66^ In Slovenia, around 11% higher iodine-131 activity was needed to eliminate hyperthyroidism after the change from mild iodine deficiency to adequate iodine supply.^68^

Iodine deficiency is associated with an increased susceptibility of the thyroid gland to nuclear radiation and with an increased risk of developing radiation-related thyroid cancer.^32,33,64^ Although data on iodine intake at the time of the Chernobyl catastrophe are not available, the region had historically been known as an area of iodine deficiency.^70^ Additionally, research conducted in the affected territories during the first decade after the disaster also pointed to the problem of iodine deficiency, with some areas placed even in the category of severe iodine deficiency.^71^ An epidemiological study in the Russian Federation confirmed that the risk of thyroid cancer was significantly associated with thyroid radiation dose and inversely associated with urinary excretion levels.^72^ In severely iodine-deficient areas, the risk of radiation-related thyroid cancer was approximately 2**–**3 times higher than in areas with adequate iodine intake.^72,73^ Ensuring an adequate supply of iodine is therefore an important measure to reduce the risk of exposure to the harmful effects of radioactive iodine at the population level.^64^

In the Fukushima nuclear disaster, long-term dietary habits with high iodine content, mostly from seaweeds, certainly contributed to a lower radiation burden on the thyroid glands of the exposed population.^33,74^ Based on available data from dietary records, food surveys, urine iodine analysis, and seaweed iodine content, it was estimated in 2011 that the average iodine intake in Japan exceeded 1000 μg/day.^75^ Additionally, a study of children performed over a 5-year period after the accident confirmed sufficient iodine intake, with urine iodine content being twice the limit recommended by the WHO.^74^

## Thyroid blocking with potassium iodide administration

Timely administration of stable iodine is highly effective in reducing radiation exposure to the thyroid.^32^ It saturates the thyroid, inhibiting NIS activity, and consequently blocking the uptake of radioactive iodine into the thyroid.^33^ Inhibition of I^−^ uptake appears to occur within a few hours after exposure to I^−^ excess.^76^ Early animal and *in vitro* studies demonstrated that after acute I^−^ exposure, NIS mRNA levels decreased within 6 hours, while NIS protein levels decreased only after 24 hours, indicating that the reduced NIS expression does not account for the initial I^−^ uptake inhibition.^31,69,76^ Subsequent research demonstrated that acute excess of I^−^ leads to NIS inactivation at the plasma membrane, caused by reactive oxygen species generated in response to elevated I^−^ levels.^76^ An excess of stable iodine also leads to the displacement of radioiodine at the carrier site on the basolateral membrane, inhibiting its entry into the cells.^77^ In human investigations, it was found that single doses of sodium iodide exceeding 10 mg suppressed 24-hour thyroid radioiodine uptake to approximately 1%, while continued daily intake of 15 mg or more consistently yielded values below 2%.^78^

For thyroid protection in nuclear emergencies, the most commonly used form of stable iodine is potassium iodide (KI) tablets, where 130 mg of KI contains 100 mg of iodine.^32,79^ The WHO recommends thyroid blocking when the estimated thyroid radiation dose exceeds 0.05 Gy. This protection is suitable for adults under 40, given the higher prevalence of thyroid diseases in older individuals, where the risks of excessive iodine intake outweigh the potential benefits. WHO advises a single administration of 130 mg of KI for adults, adolescents, as well as pregnant and breastfeeding women. For children aged 3–12 years, the recommended dose is 65 mg, for children aged 1 month to 3 years it is 32 mg, and for infants under 1 month old it is 16 mg.^79^ Iodine is quickly and almost entirely absorbed in the stomach and duodenum.^24^ KI tablets offer protection for approximately 24 hours. If exposure persists beyond this timeframe, repeated administrations for up to 7 consecutive days may be required for certain groups, excluding neonates, pregnant or breastfeeding women.^79,80^

KI tablets offer effective protection only within a narrow time window less than 24 hours before and up to 2 hours after exposure.^32,79^ They are 99% effective when administered at the time of exposure, at least 85% effective within 24 hours before or 2 hours after, but ineffective 96 hours before and only 50% effective 3–4 hours after.^32^ However, administration later than 24 hours following exposure can even be harmful, as it can lead to the trapping of radioactive iodine in the thyroid, thereby prolonging its biological half-life and increasing its harmful effects.^79^ Due to the narrow time window, pre-distribution of KI tablets in exposed areas, such as the vicinity of nuclear reactors, is important.^34^

During the Chernobyl accident, administering KI to 95% of Polish children and 23% of the total population was estimated to reduce their projected thyroid dose by approximately 40%.^34,81^ In Belarus and Russian children under 15 years of age, administering lower doses of KI primarily to prevent goiter reduced the risk of radiation-related cancer by 3-fold.^73^ However, Japan did not implement KI prophylaxis for the general public after Fukushima accident, acknowledging its unpreparedness for such measures.^32^

Thyroid blocking with KI may be associated with adverse events. Based on observations from Poland, mild reactions, such as skin rash, vomiting, or abdominal discomfort were experienced in less than 4% of children and less than 3% of adults.^32,33^ In neonates, the concern can be iodine-induced hypothyroidism, which can occur even with iodine administration exceeding twice the recommended amount.^81^ In adults, however, excess iodine exposure can induce thyroid dysfunction in patients with thyroid autoimmune diseases or goiter.^34,82^ Since these thyroid diseases are prevalent in the population and their incidence rises with age, the administration of KI tablets is associated with health risks, particularly after the age of 40.^34,83^ Finally, it is important to note that patients who have had a thyroidectomy or are undergoing hormone replacement therapy for other reasons do not need protection with KI tablets.^34^

## Conclusions

Given the threat of nuclear accidents, good preparedness is crucial for effectively managing critical events. One of the many products of nuclear fission is radioactive iodine, which, due to its properties, can contaminate the broader area surrounding the accident. Adverse effects from exposure to I-131 depend on several factors, including national-level emergency preparedness and response, the thyroid dose received, the age of the exposed person, iodine intake prior to exposure, the adequacy of KI tablet administration, and any pre-existing thyroid disorder (Table 2). Experience from past accidents indicates that children's thyroids are the most vulnerable. The risk of thyroid cancer starts to increase a few years after exposure and is related to the thyroid dose received, with higher risks observed even many years later. However, in older adults, the risk of adverse effects from I-131 is lower, yet the prevalence of thyroid diseases is high. Therefore, the use of KI tablets for thyroid blockade could pose health risks. The most effective measure to reduce the consequences of exposure to radioactive iodine at the population level is ensuring adequate iodine intake, which is achieved in several countries through the consumption of iodized salt.

**TABLE 2.** Influential factors on the risk of harmful effects from iodine-131 in nuclear accidents

**Parameter**	**Higher risk**	**Lower risk**
**Exposure**	Late public notification Accompanying accident (earthquake, fire …) Exposed workers	Early public notification Preventing contaminated food and water intake Indoor sheltering
**Received dose (Gy)**	> 0.05	< 0.05
**Age**	Children (especially < 5) Exposure in utero	Adults
**Iodine intake before exposure**	Deficient	Sufficient
**Thyroid blocking (KI tablets)**	No blocking Inappropriate timing	Appropriate timing (less than 24 hours before and up to 2 hours after exposure)
**Pre-existent thyroid disease**	Iodine deficiency disorders No pre-existent thyroid disease	After thyroidectomy Hormone replacement therapy for other reasons
**Medical surveillance**	No surveillance	Close surveillance in high-risk individuals
